# The Manner of Using Quinine

**Published:** 1846-12

**Authors:** 


### PART V.—ABSTRACTS.

### ARTICLE XVII.

### THE MANNER OF USING QUININE.

Any recent experience in what may be called the new method of using this important remedy is possessed of great interest. Dr. R. S. Holmes of the U. S. A., has a paper in the American Journal of Medical Sciences for October, 1846, on "Quinine and Malaria," in Florida, from which we gather the following practical items.

In intermittents he never gave it when the bowels were in a torpid or gorged condition, its effects then seeming to be lost. Has given it when the bowels were actively purged or irritated, combining it with laudanum. In an ordinary intermittent, after having freely opened the bowels, fifteen grains were generally given at one dose, on the evening of the day on which the purge was taken. If the fever was a quotidian, the paroxysm recurred on the next day, remaining as long, but of a lighter grade; on the third day the patient was free from disease. If the disease was of the tertain form there would not be a paroxysm after the administration of the quinine, from which Dr. H. deduces that it requires about eighteen hours for the full effects of quinine to manifest themselves, and which his subsequent practice fully verified.

He did not find this agent to become less active by repetition, having given the same number of grains time after time for a successive attack, and always with the desired result.

The quinine never was given during the fever or chill, on account of the difficulty of retaining it, not that he believed that if it was retained, it would have been injurious or have operated less effectually.

In congestive fever, two indications are to be met immediately,—to rally the patient for the time being by friction, mustard poultices, and stimulants, and to prevent the periodical return. In the first few minutes after seeing the patient, has given him from thirty to sixty grains of quinine at one dose, n water or brandy; the brandy for the former, and the quinine for the latter indication. If the attack was severe, and the quinine not retained, it was given by enema.

In the writer's own words,—"I have given the quinine in the highest stage of the fever, and in the lowest of prostration; never permitting any existing state of inflamed bowels or stomach to deter from its administration, or lessen the dose in which I would otherwise have given it; I have not lost a patient from inflammation following congestion, and where the quinine has been given as here mentioned. I have not seen a patient die, who survived twenty-four hours from the time of attack. The fatal cases are those in which the quinine has not been given in a proper quantity, or where it has not been thrown into the constitution for a sufficient length of time to reach the disease."

After referring to the opinion, that quinine, in large doses, produces excitement in the brain, and irritation of the bowels; and therefore that even in urgent cases of congestive fever, where these organs are implicated it is sometimes withheld, for fear of aggravating these morbid conditions! quotes a case to illustrate the benefit of quinine, where such complications exist.

It was a dangerous remittent, with inflammation and congestion of the stomach and intestines of a high grade, so great that the attending physician directed his efforts to the subduing of the abdominal disease, and being at the same time impressed with the idea of the irritating power of quinine, the fever was suffered to proceed unchecked. On the seventh day, it was believed that the patient would not live forty-eight hours, if the fever remained at the height which it then reached, whilst it was supposed that the intestinal inflammation, would not prove fatal in less than three or four days.

Therefore, with much hesitation, forty grs. of quinine were given in two doses in twenty-four hours. The next exacerbation was trifling, and afterwards did not occur. The severe and obstinate inflammation was recovered from in six weeks.

The following points of practice are thus set forth:—

"Every periodical disease is to be checked immediately. Quinine, as a remedy for periodicity, is to be given regardless of any existing state of inflammation. Never give quinine in divided doses, when directed for the immediate cure of a periodical disease. To be certain of the operation of quinine in a constitution with which you are not acquainted, it must be given eighteen hours before the desired result. In emergent cases, it may be given in the lowest state of prostration, or the highest grade of the fever. As a general rule, fifteen to twenty grains will be necessary for an intermittent, and thirty to fifty, for a congestive fever. Never give quinine for the cure of a periodical disease in anticipation, when the periodicity exceeds five days."

In larger doses than tonic ones, its tonic action is quickly followed by stimulation, and this again by sedation: but to produce this last in any great degree, it must be given in large doses. Consequently if it is given immediately before a chill, and arrests it, it does so by its stimulating power; but this stimulation is not so certain, or effectual, as its sedative effect, in which consists the great anti-periodic power of quinine.

Dr. Wm. M. Boling, of Montgomery, Ala., considers quinine as a prompt, decided, unequivocal sedative of the heart and arteries, applicable in all cases of remittent fever, accompanied by an increase in the *force* and frequency of the circulation, and "the very best remedy for bringing about that absence of great febrile excitement, which was considered requisite for its successful administration as an anti-periodic." In inflammatory cases, when the pulse indicates it, blood-letting is recommended in conjunction with the quinine, but not as a preparative.

In reference to purgatives he advises that moderate evacuations from the bowels be procured before giving the quinine, as thereby it acts more decidedly, and less of the remedy is required, more than this is not considered beneficial.

It is the author's opinion that much mischief is done in postponing the administration of quinine by a preparatory treatment, in order to procure a more perfect remission: severe cases dying while under this preparation, and mild cases becoming worse; and of whatever type or character the (remittent) fever may be, if the case is dangerous, no time is to be lost in bringing the system decidedly under the use of quinine. Gives it in the remission when this is well marked and of some duration, probably more from habit than anything else, but when the case is urgent gives it without regard to the stage of the paroxysm. "Two portions of from eight to sixteen grains each, according to the urgency of the symptoms given, within a couple of hours of each other, will most generally bring the patient under its influence, two or three hours after the administration of the second portion." Where the case is very violent, with but a short period, until the next exacerbation, the whole may be given at once.

After the system is fully under the influence of the medicine, eight grains every third or fourth hour, will produce all the good that can be obtained from it.

When there is an inflammatory complication, the remedy must be continued until this is subdued. If withdrawn too early, the local inflammation will increase.

Under the favorable action of quinine the pulse becomes less frequent, if it has been small and corded, it is increased in volume, and if hard and firm, is rendered soft.

The skin becomes moist and cool, the tongue moistens and the thirst diminishes.

In the pernicious or congestive remittents, we must be cautious in our administration of quinine. Here, where there is much real as well as apparent debility, if we do not give sufficient to arrest the disease at once, the patient may die in the next exacerbation, whilst at the same time, "it is to be dreaded that a quantity necessary to effect this, may destroy by its depressive action." If, as is often the case, the state of the stomach and bowels forbid the use of stimulants, opium is the best remedy to sustain the system against the depressing effects of quinine, whilst at the same time it controls the irritability of the stomach.

When the stomach is so very irritable as not to retain the medicine, even when thus guarded by opium, it is to be administered by enema,—twenty to thirty grains, every second hour, in two ounces of starch, until the desired effect is produced. "As a stimulant, the opium may be administered in the same way, as much as the patient can bear without narcotism." When diarrhœa exists, or when the rectum has been rendered irritable by laxative enema, 80 to 100 drops of laudanum may be injected in a little starch, previous to the quinine.

The existing impression that mischief must result from giving quinine, except during the remission, is unfounded. "So far is this from being true, that there is nothing with which we can more effectually aid in cutting short an exacerbation, than the quinine itself, commenced, and administered freely during that time." See Boling on remittent fever, *Am. Jour. Med. Sci.* July, 1846.

The experience of Dr. J. J. B. Wright, U. S. A., in Arkansas and Florida, with quinine in remittent fevers, is substantially the same as that already noted, as the following quotation will show.

"They who contend that its curative agency is due to a direct effect on the tonicity of the muscular fibre, would prescribe its use in all cases where this vital property, or the contractibility of the muscular system, might be presumed to be in an exalted position. But the physician experienced in diseases of southern climates will tell you that he is in the practice of administering it when the condition of these vital properties is, seemingly, at least, above par. For instance, he exhibits it at the very height of the paroxysm of the remittent fever of his climate, and finds as the result of its action, a reduction in the force and frequency of the pulse,—a diminution of animal heat,—a moist condition of the skin,—a subsidence from febrile disturbance to fair convalescence. Now, if the experience and observation are right, can the theory be otherwise than wrong? On the other hand, those who maintain that the remedy acts by sedation, do not hesitate to exhibit the article when the powers of life are depressed to the utmost limit compatible with existence; and they aver that its agency is curative under these circumstances. True it is that the writer has himself frequently given the quinine in both of the conditions stated, and in both his experience teaches that advantage resulted from the practice. He has witnessed a decided improvement to follow the exhibition of ℈ij. of quinine, repeated in two hours, in advanced congestive fever, when the condition of the case was characterized by a lethargic state of the sensorial functions, verging on coma,—cold extremities,—cool surface, bathed in limpid perspiration,—dry and pallid tongue,—feeble and fluttering pulse, &c. And again, whilst on duty in Florida, in the summer of 1842, in charge of the General Hospital, it was his usual custom, after attentive observation of the safety of the plan, to exhibit twenty grains of quinine *at any period of the paroxysm* of the remittent fever of that country, and he is safe in declaring that the practice was successful—as the Quarterly Report to the Surgeon General's Office will testify, not a single death from remittent fever having been reported during the season, nor from its sequelæ."—Wright on the use of Sulphate of Quinine, *N. Y. Journal of Med. and Collat. Sciences.* September, 1845.

Dr. W. H. Van Buren, late of the U. S. A., furnishes concurrent testimony of this mode of using quinine, in Florida.

In simple intermittents of every variety, he gave it in doses of from 15 to 20 grains, from six to twelve hours before the chill, and, where there was no visceral or glandular disease, used no preparatory treatment.

In remittent fevers, the paroxysm being treated according to the particular indications, "The moment the skin becomes moist, without particular regard to the state of the pulse, I give from 20 to 25 grs. quinine, according to the severity of the case," &c., and repeat it in from four to eight hours. When the stomach rejected it, applied 60 or 80 grs. to a blistered surface on the epigastrium. In malignant intermittents found the same doses equally successful, but it was necessary to keep up its action for a longer period than in simple remittents.

In the congestive fever, had no precise limit to the use of the remedy, but believes that 40 grs. if retained and digested will suffice, combined with stimulants, internally and externally; under this treatment, considers the disease perfectly manageable, when it is applied in time by a physician of judgment.

In yellow fever, has never seen "any decided and permanent good effects from the use of quinine," though "employed in doses of every size in a number of cases," the writer believes "that the primary seat of the disease in yellow fever, is in the *blood*, and not in the nervous system, as in miasmatic diseases. Derangement of the nervous system being the *consequence*, and not the *cause* of the disease." By this he wishes to "explain if possible," the inefficiency of quinine in the cases referred to. Van Buren's Report on the use of Quinine. *N. Y. Jour. Med.* Jan., 1846.

Abundant evidence has been adduced to establish the method of giving quinine, which the papers referred to advocate, in the *South*. But it still remains to be seen how far the same rules apply to the *North*. That it does not apply to all the portions heard from would seem to be proved, whilst in other localities the Southern practice, with certain modifications, can be followed with advantage.

Thus Dr. Geo. Mendenhall, of Cleveland, Ohio, found venesection, local bleeding, emetics, and revulsives generally useful. Diaphoretics were "almost always necessary," and cathartics indispensable. The following extract will give an idea of the practice pursued.

"As a general rule, a depletory and cathartic course was necessary. These, with the other means which have been mentioned, had to be continued until a decided remission or intermission in the febrile action should take place, unless the system became too much reduced to bear them further. When the periodicity was well marked, and the tongue began to clean, quinine could be borne, and almost invariably with advantage. The quantity which we have been in the habit of giving during an intermission or distinct remission, was from ten to fifteen grains, dividing it into about five equal portions, and watching the effects of each dose (which were given about two hours apart,) until after the administration of the first two or three. When perspiration followed its use there could be no question of the propriety of its exhibition, and the result was then in all cases satisfactory." Mendenhall on Quinine in Fevers. *Am. Jour. Med. Sc.*, July, 1846.

Dr. Austin Flint, of Buffalo, has advocated the employment of quinine in large doses at that place. His practice in intermittents was to give from three to five grs. at a dose, repeated every one or two hours, rarely exceeding twenty grains in the twenty-four hours; and in general principles of his treatment for intermittents and simple remittents, coincides with the southern practice. Flint on the employment of quinia in large doses. *N. Y. Jour. Med.*, March, 1846.

Dr. Mendenhall in his paper from which I have quoted, objects to the general application, at the north, of Dr. Flint's practice on the ground "that nearly or quite all the cases which he (Dr. F.) treated so successfully with large doses of quinine without preparation, contracted the disease at some other place, and that in their removal to Buffalo they were removed away from the 'continued, concentrated action of malaria.' In these cases, then, one curative means was complied with, which of itself is often sufficient to accomplish a cure, viz.: a removal from the cause of the disease."

Now, how far this was the case cannot here be decided. But I have had, during the past season, the evidence of my own, and other physicians' practice, that quinine can be given in large doses to arrest intermittents and remittents, with entire success. The form of remittent which we have had in Chicago and its vicinity, is that popularly called "Chill Fever:" there was generally during the remission an entire absence of heat of skin, the pulse remaining frequent though soft, the general pain and soreness in the severer cases also continuing. A common accompaniment was irritability of the stomach, so that in some cases, during the exacerbation, not even cold water in small quantities was retained. In the severer cases, the patients were delirious whilst the fever continued.

Usually, the only preparative treatment was a full dose of oil, after a small portion of calomel, the former given usually during the remission, as often the stomach would have rejected it at any other period. I generally found the remission more complete after the bowels had been emptied. In the next remission, gave about 20 grs. quinine with two or three grs. powd. opium, in four doses every two hours, often in two or three doses, and occasionally all at once, when I had not sufficient time to divide it before the accession of the chill.

I, in perfect confidence, anticipated the suspension of the disease, if not at the next exacerbation, certainly at the time of the second, and was very seldom disappointed. It occassionaly was not arrested until the latter period, verifying Dr. Holme's observation that it requires about eighteen hours for the sedative effects of the remedy to be developed.

The usual adjuvants of cold to the head, warmth to the feet, diaphoretics, sinapisms, and blisters, were occasionally employed during the exacerbation, and the quinine was sometimes delayed a day or two to allay a very irritable stomach, but still the principal agent was the quinine, and I have often given it in remittents, with a dose of calomel without any preparation, and have never known any ill effects to arise from it.

As County physician, I had very many such cases among the poor population, most of whom were in an unfavorable situation as regards nursing, and all the ordinary comforts of sickness.

Here the experience of Dr. Mendenhall on the one side, and Dr. Flint, and some of us of Chicago, on the other, does not agree as to the best mode of using quinine in the North.

It is possible that peculiarities in the locations of the places, modify the disease. As it is, the subject requires a more extended observation in the northern portion of the country, which it doubtless will ere long receive.

